# Dr. Girdlestone's Case of Fracture

**Published:** 1802-01

**Authors:** T. Girdlestone

**Affiliations:** Yarmouth


### *To the Editors of the Medical and Physical Journal.*

GENTLEMEN,

IF you think the following case worth the insertion in your useful production, it is much at your service.

From your, &c.

*Yarmouth, Dec.* 8, 1801. T. GIRDLESTONE, M. D.

---

On the 22d of March, 1795, Mr. Aldred, a young man of about 22 years of age, by a fall from a horse, received a fracture on the upper part of the occiput.

Mr. Darby, of Lowestoff, was his surgeon, who called in his brother surgeons, Mr. Arnold and Mr. Glasspoole, and by their advice two Yarmouth surgeons were added to the consultation, tation, Mr. Downes, and the late Mr. Francis Turner, who is ftill gratefully recollected in this town and neighbourhood for his fuccefs in many cafes of bubonocele, depreffion of cataracts, and other furgical operations. Thefe gentlemen endeavoured to trace the fracture without being able to difcover its extent, till they had approached, as near as they thought it prudent, the foramen magnum. Several veffels were wounded, and much blood was loft by the refearch, before the wound was dreft. The next day I accidentally paffed through the town, and was defired to vifit this patient. He was then alternating between convulfion and ftupor, with a pulfe in a conftant ftate of interruption. His motions and urine paffed away involuntarily, and his food was obliged to be poured down his throat in a liquid ftate.

Without any hopes of his recovery, either of life or intellects, I ordered him fudorofic dofes of antimony and opium at intervals.

Under thefe fymptoms he continued nearly ftationary in every refpect, except that of the fracture gradually healing, till about the fourth week. About this time the pulfe become regular, and the fenfes gradually to return fo completely, that the ftrongeft light or found could be borne without any inconveniency. But his character had undergone a very alarming revolution. From being naturally an over-anxious-minded young man, he become voluptuous, fprightly, and unthinking.

In this ftate he continued till between the 8th and 9th week from the accident, when his original character re-appeared and has ever fince remained.

P. S. In page 492 of the laft Medical Journal, the Editors afk the following queftion.

"Can Typhus be fo frequent at this feafon of the year?"

As far as ten years experience in one large town and neighbourhood tends to folve the queftion, I can affure the Editors that the greateft number of Typhus Fevers in this diftrict, has generally prevailed from the latter end of October till the firft, fecond, or third week in December, when the number of Typhus Fevers, as well of Scarlatina, (if that difeafe had been endemic,) generally leffened.*

---

* See Addrefs to Correfpondents at the end of this Number.

